# Supplementary material for: Maternal Hypermethylated Genes Contribute to Intrauterine Growth Retardation of Piglets in Rongchang Pigs
Source: Int J Mol Sci. 2024 Jun 12;25(12):6462. doi: 10.3390/ijms25126462 (PMC11203632; doi:10.3390/ijms25126462)
Supplement: Supplementary file 1 [file ijms-25-06462-s001.zip › Supplementary Figures.pdf]

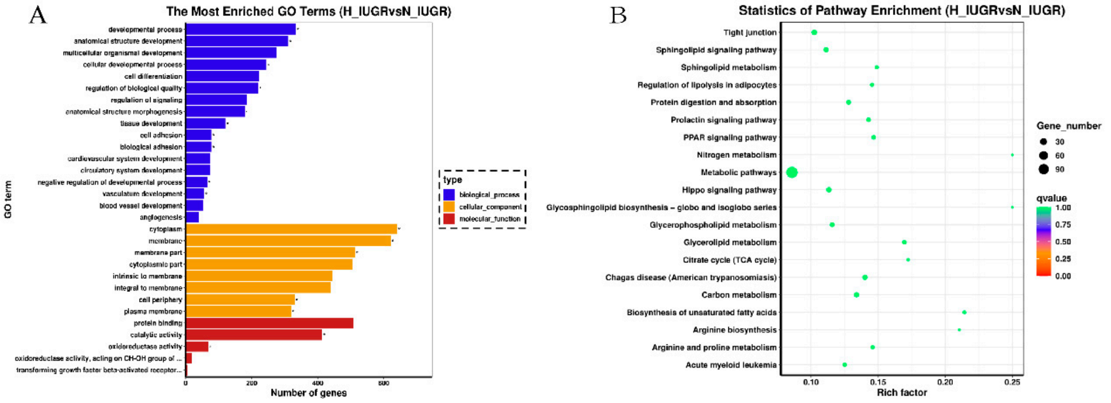

Figure.S1 Enrichment Analysis. (A) GO term enrichment analysis of DEGs, with the 30 top terms obtained by GO enrichment. The x-axis represents the number of DEGs in the term, and the y-axis represents the enriched GO terms. The top 30 GO terms included 20 terms for biological process, 9 terms for cellular component and 1 term for molecular function. "*" indicates p<0.05. (B) Enriched KEGG pathways (top 20) for the DEGs that were obtained by KEGG enrichment. The size of the dots indicates the number of expressed genes in the pathway, and the color of the dots represents the different Q value ranges of the pathway.

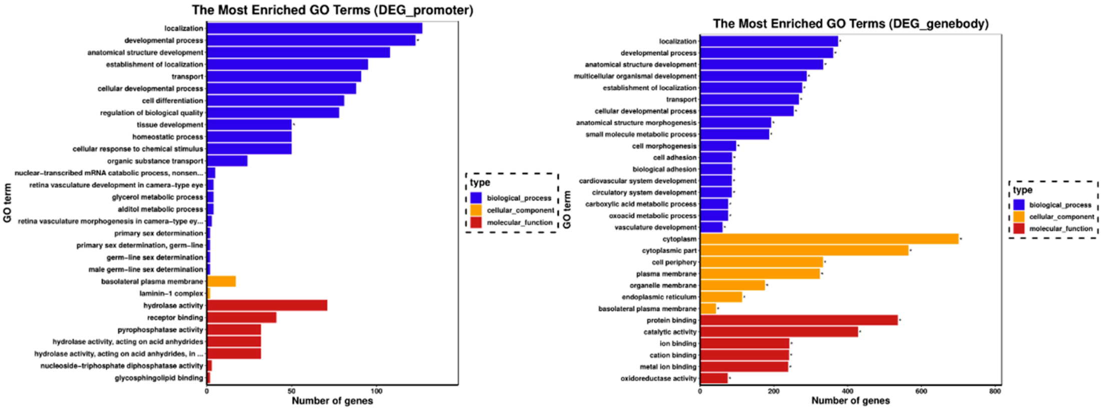

Figure.S2 GO enrichment analysis of methylated genes and differentially expressed genes in the promoter region (left); GO enrichment analysis of methylated genes in the body region and differentially expressed genes(right).
