# Supplementary material for: Maternal Hypermethylated Genes Contribute to Intrauterine Growth Retardation of Piglets in Rongchang Pigs
Source: Int J Mol Sci. 2024 Jun 12;25(12):6462. doi: 10.3390/ijms25126462 (PMC11203632; doi:10.3390/ijms25126462)
Supplement: Supplementary file 1 [file ijms-25-06462-s001.zip › Supplementary Tables.pdf]

**Supplementary Table 1** Sequencing data quality and bisulfite conversion efficiency

| Sample | Raw Reads | Clean Reads | Q20（%） | Q30（%） | GC（%） | BS（%） |
|---|---|---|---|---|---|---|
| H_IUGR1 | 277413067 | 258179035 | 96.95 | 90.93 | 21.98 | 99.824 |
| H_IUGR2 | 274458003 | 256119180 | 96.65 | 90.33 | 21.58 | 99.833 |
| H_IUGR3 | 276279424 | 259469627 | 96.70 | 90.40 | 21.25 | 99.839 |
| H_IUGR4 | 257812810 | 240868231 | 96.80 | 90.56 | 21.32 | 99.825 |
| H_IUGR5 | 281581126 | 264045532 | 96.90 | 90.76 | 21.23 | 99.837 |
| N_IUGR1 | 267708204 | 249833027 | 97.00 | 91.04 | 21.37 | 99.811 |
| N_IUGR2 | 324300567 | 305398522 | 96.60 | 90.03 | 20.84 | 99.788 |
| N_IUGR3 | 315352583 | 298611072 | 96.76 | 90.42 | 21.18 | 99.729 |
| N_IUGR4 | 286523065 | 268830015 | 96.95 | 90.87 | 21.39 | 99.838 |
| N_IUGR5 | 300913999 | 275711123 | 96.07 | 89.14 | 21.13 | 99.820 |

**Supplementary Table 2: Primers for RT-qPCR**

| Gene | Primer sequences (5’to 3’) | TM(℃) | Product size(bp) |
|---|---|---|---|
| ANXA1 | F:CTCGATTGCACTGAGGATCA<br>R:GCTGATTCTGGCCACTTCTC | 60 | 116 |
| SPOCK1 | F:GGGCTGGATGTTCAACAAGT<br>R:CTCCAGGTTTCTTGGAAGCAG | 60 | 105 |
| CPS1 | F:AAGTCCTGGGGACCTCAGTT<br>R:AGGACAATGCCTGAGCCTA | 60 | 113 |
| FOSB | F:GTGAAGTTCAAGTCCTCGGC<br>R:TCACAGAGCAAGAAGGGAGG | 60 | 120 |
| IGF2 | F:ATCGTGGAAGAGTGCTGCTT<br>R:CATAGCGGAAGAACTTGCCC | 60 | 114 |
| UCP2 | F:GCCAACAGACGTGGTAAAGG<br>R:TGGCATTACGAGCGACATTG | 60 | 96 |
| ITGA8 | F:CTTTCACATACCTGCGGCTC<br>R:TCAAAGGGTATCTGCCTGCA | 60 | 154 |
